# Phenolic Evolution During Extended Skin Contact: Effects of Harvest Maturity on “Orange” Wine Composition and Color

**DOI:** 10.3390/foods15183334

**Published:** 2026-09-20

**Authors:** Bettina-Cristina Crișan (Buican), Camelia Elena Luchian, Elena Cristina Scutarașu, Laurian Vlase, Lucia Cintia Colibaba, Ana-Maria Vlase, Cătălin Ioan Zamfir, Stamatina Kallithraka, Valeriu V. Cotea

**Affiliations:** 1 “Ion Ionescu de la Brad” Iași University of Life Sciences, 3rd M. Sadoveanu Alley, 700490 Iași, Romania; bettina.buican@iuls.ro (B.-C.C.); cristina.scutarasu@iuls.ro (E.C.S.); cintia.colibaba@iuls.ro (L.C.C.); catalin.zamfir@acadiasi.ro (C.I.Z.); valeriu.cotea@iuls.ro (V.V.C.); 2 Department of Pharmaceutical Technology and Biopharmaceutics, University of Medicine and Pharmacy, 8 Victor Babes Street, 400347 Cluj-Napoca, Romania; 3 Department of Pharmaceutical Botany, Faculty of Pharmacy, Iuliu Hatieganu University of Medicine and Pharmacy, 8 Victor Babes Street, 400347 Cluj-Napoca, Romania; gheldiu.ana@umfcluj.ro; 4 Romanian Academy–Iași Branch, Research Center for Oenology, 9H, M. Sadoveanu Street, 700490 Iași, Romania; 5 Laboratory of Enology and Alcoholic Drinks, Department of Food Science and Human Nutrition, Agricultural University of Athens, 75 Iera Odos, 11855 Athens, Greece; stamatina@aua.gr

**Keywords:** phenolic extraction evolution, orange wine, winemaking, maceration time, harvest timing

## Abstract

Extended skin contact transforms white wines into polyphenol-rich “orange” wines, yet the optimal combination of harvest maturity and maceration time is not fully established. The evolution of physicochemical, phenolic, and chromatic properties was evaluated in Sauvignon blanc and Fetească regală wines produced with 1, 3, and 6 months of skin contact at technological and advanced phenolic maturity. Phenolic compounds were quantified by HPLC-DAD/MS, global indices by Folin–Ciocalteu (FCI) and total polyphenol index (TPI), and color by CIELAB. Skin-derived monomers were rapidly extracted, whereas seed-derived oligomers (procyanidins B2, C1) and ellagic acid required prolonged hydroalcoholic exposure. In phenolic-mature Sauvignon blanc, total phenolics reached 338.8 mg L^−1^ after 6 months, with total flavonoids of 312.8 mg L^−1^ and procyanidin B2 of 91.7 mg L^−1^. In technologically mature Fetească regală, flavonoids declined from 266.8 mg L^−1^ at 3 months to 201.7 mg L^−1^ at 6 months. Three-way ANOVA identified significant main effects of cultivar, harvest maturity, and maceration duration on all evaluated phenolic responses (all *p* < 0.0001), together with significant interactions; cultivar × maturity for total phenolic acids was the only nonsignificant interaction (*p* = 0.071). In technologically mature Sauvignon blanc, titratable acidity decreased from 6.16 to 4.78 g L^−1^ and pH increased from 2.98 to 3.32 between the unmacerated control and 6 months. Exploratory correlations showed inverse associations between titratable acidity and pH (r = −0.830) and between malic (MA) and L-lactic (LA) acids (r = −0.901), without implying causality. Under the conditions examined, 1–3 months of maceration generally provided substantial phenolic extraction while limiting some declines observed after 6 months, particularly in technologically mature Fetească regală.

## 1. Introduction

Driven by evolving consumer preferences for sustainable, clean-label, and minimally processed food matrices, the global enological sector is increasingly evaluating low-intervention production systems [1,2,3]. Within this paradigm shift toward process sustainability, ancestral vinification techniques are undergoing systematic scientific re-assessment. Prominent among these is the production of long-macerated white wines, commercially and colloquially designated as “amber” or “orange” wines, which have transitioned from niche artisanal products to a distinct, scientifically relevant category [4,5,6]. Conventional white vinification protocols strictly limit contact between the liquid phase and solid grape fractions (skins, seeds, and vascular bundles) to minimize the extraction of astringent or easily oxidizable compounds, restricting skin contact to brief, pre-fermentative macerations primarily intended to facilitate volatile aroma release in specific aromatic cultivars [4,7,8]. Conversely, long-macerated white wines involve prolonged solid–liquid contact extending from one month to several years. This extended duration subjects the grape skins, seeds, and occasionally stems to continuous extraction throughout the fermentation and post-fermentative phases, fundamentally altering the physicochemical equilibria and phenolic architecture of the final wine matrix [9,10].

The evolution of this extended solid–liquid extraction is primarily governed by changes in the liquid’s chemical properties [11]. As fermentation progresses, yeast converts sugars into alcohol. This increase in ethanol lowers the liquid’s polarity, turning it into a hydroalcoholic solution that dissolves plant compounds, such as hydrophobic and high-molecular-weight molecules, that do not easily dissolve in water alone [12,13]. Because of this, standard white winemaking (where grape skins are removed quickly) only extracts the water-soluble phenolic acids already present in the juice. In contrast, extended skin contact pulls a much wider range of polyphenols out of the solid skins and seeds and transfers them into the wine [14]. This newly established polyphenolic architecture serves as the primary determinant of both the technological and organoleptic quality attributes of the final beverage, offering producers a mechanism to modulate specific product profiles. Technologically, these extracted polyphenols govern the physicochemical stability and aging potential of the wine matrix, while simultaneously mediating defining sensory characteristics such as color, hue, clarity, bitterness, and astringency [15].

Long-macerated white wines bridge white and red wine chemistry through significant flavonoid enrichment [9]. Unlike conventional white wines dominated by juice-derived hydroxycinnamic acids, long-macerated varieties incorporate skin-derived flavonol glycosides (quercetin, kaempferol, and isorhamnetin) that stabilize yellow-orange pigments via co-pigmentation [15]. Additionally, extended skin and seed contact extracts substantial quantities of flavan-3-ol monomers, specifically (+)-catechin and (−)-epicatechin, and condensed tannins [16,17,18]. Over time, these polyphenols undergo continuous rearrangements, including acid-catalyzed cleavage, subunit reorganization, and oxidative polymerization [4,19].

The extraction potential during extended maceration is fundamentally influenced by the grape maturity parameters established at harvest, requiring a precise alignment between technological maturity (soluble solids accumulation and titratable acidity) and phenolic maturity. Optimal phenolic maturity ensures maximum accumulation of skin-bound flavonols alongside structural lignification of the seed coat, which reduces the extractability of harsh, monomeric seed tannins during prolonged hydroalcoholic exposure [20,21]. Aligning these maturity indices allows winemakers to tailor contact duration specifically to the grape’s chemical profile, avoiding over-extraction while maximizing phenolic integration.

Precision control over the structural, sensory, and phenolic profiles of wine depends fundamentally on managing the contact duration at the solid–liquid interface. Because skin- and seed-bound phenolic fractions extract at distinct rates as the hydroalcoholic solvent evolves, time-resolved monitoring is essential to optimize color density, body, and astringency while preventing excessive bitterness. Transitioning from empirical winemaking practices to a predictive, engineering-based model requires quantitative data detailing how distinct phenolic fractions and matrix parameters shift throughout maceration. To establish this baseline, this investigation systematically profiles the continuous physicochemical evolution of extended skin contact vinification across multiple temporal intervals.

The aim of this paper is to evaluate how the physicochemical and phenolic characteristics of Sauvignon blanc and Fetească regală wines varied according to cultivar, grape maturity at harvest, and maceration duration, including the possible interactions among these experimental factors.

## 2. Materials and Methods

### 2.1. Plant Material

Biological matrices comprised *Vitis vinifera* L. *cv.* Sauvignon blanc (SB) and Fetească regală (FR) grapes harvested manually during the 2023 vintage, at two distinct development stages: technological maturity and advanced phenolic maturity. The experimental material was sourced from the Iași-Copou vineyard, located within the Iași wine region of Northeast Romania (47°10′ N latitude, 27°35′ E longitude). Rigid sanitary selection was applied in the field to ensure only intact, undamaged clusters were processed.

**Figure 1:**
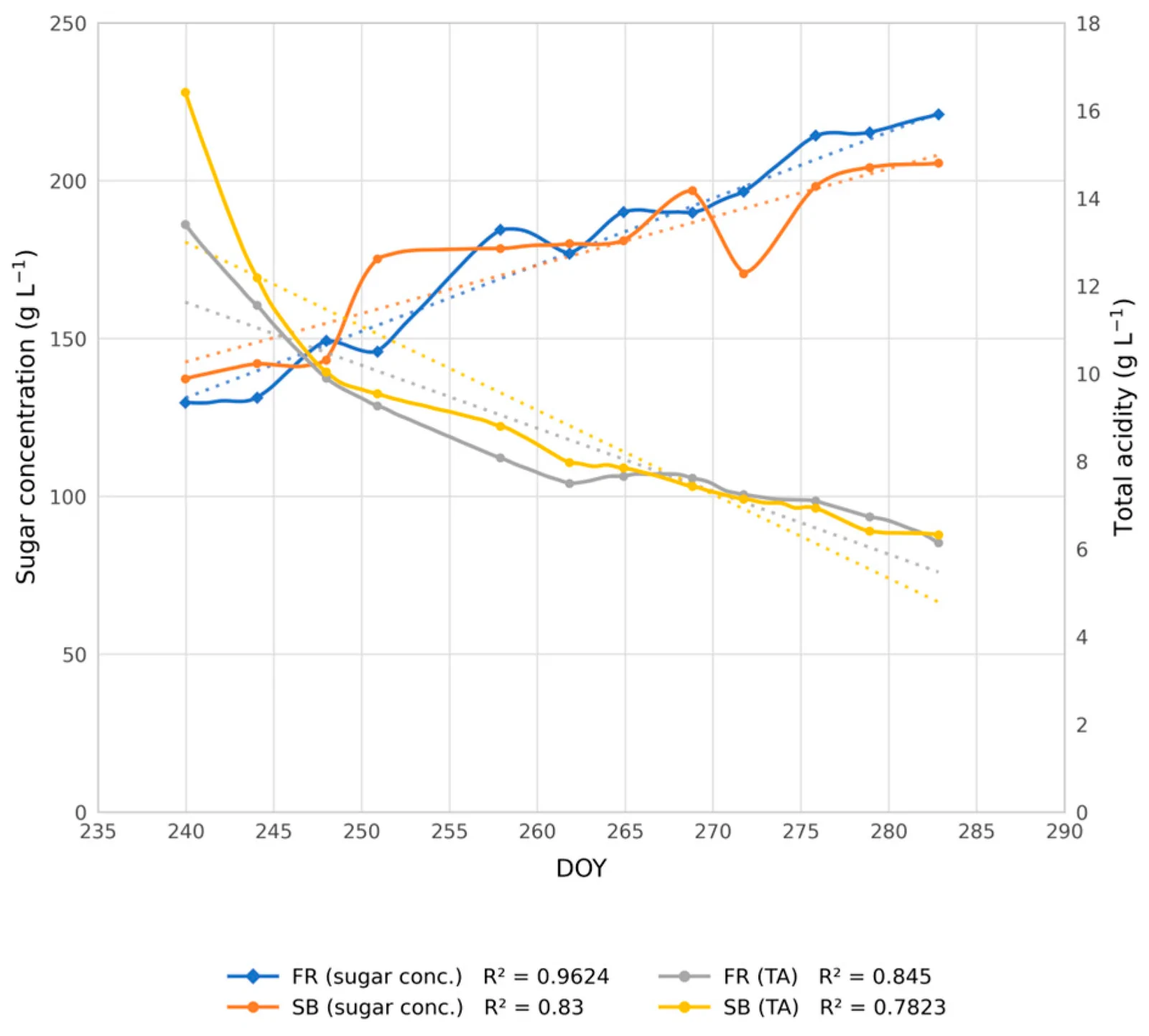
Evolution of grape sugar content and total acidity during ripening until harvest. The dotted lines represent linear trendlines for the corresponding data series.

To establish advanced phenolic maturity, vineyard sampling was conducted every 3 days by a consistent assessor to score berry pulp softness, skin tissue friability, skin tannin intensity, and seed coat brittleness. This sensory profiling was paired with continuous measurements of sugar accumulation, titratable acidity (Figure 1), and 100-berry weight to pinpoint optimal harvest timing. At harvest, initial sugar concentrations were 205 g L^−1^ for SB and 220 g L^−1^ for FR, yielding two structurally distinct matrices for subsequent extraction evolution.

### 2.2. Winemaking

The complete experimental matrix comprised 18 wine samples, including the respective controls for both grape varieties, as detailed in Table 1. Grape clusters were mechanically destemmed and crushed, and the resulting must was immediately treated with 5 g hL^−1^ potassium metabisulfite to limit premature oxidation and microbial spoilage. After SO_2_ addition, the must was thoroughly homogenized. For maceration treatments, the liquid phase and solid grape pomace (skins and seeds) were mixed at a fixed 3:2 (*v*/*v*) ratio and distributed into identical 27 L stainless steel fermentation tanks. Control treatments (SBMDC, SBMFC, FRMDC, and FRMFC) were pressed immediately after crushing to separate the juice and vinified as conventional white wines without skin contact. Post-fermentation pressing controls (SBMFCP and FRMFCP) were separated from the pomace immediately after alcoholic fermentation.

Alcoholic fermentation was initiated under uniform microbiological conditions by inoculating all vessels with the commercial *Saccharomyces cerevisiae* strain Zymaflore X5 (Laffort, Bordeaux, France) at 20 g hL^−1^, following the manufacturer’s rehydration protocol. All fermentations were carried out in a temperature-controlled room maintained at 10–12 °C. In maceration treatments, the floating skin cap was submerged twice daily by manual punching down (pigeage) to ensure continuous contact between the liquid and solid phases. Maceration was maintained for 1, 3, or 6 months. At the end of each maceration period, wines were racked, stabilized, and filtered to ensure identical baseline conditions across treatments, all maceration variants and non-macerated control wines received identical sulfiting treatments, adjusting free SO_2_ to a target concentration of 45 mg L^−1^ via potassium metabisulfite, and were subjected to the exact same post-bottling storage period. Wines were bottled in 0.75 L glass bottles under inert gas and stored until analysis. All maceration variants and control wines were produced in triplicate (*n* = 3).

### 2.3. Physicochemical Parameters

The classical enological parameters of grape musts and experimental wines were determined in accordance with the official methods of the International Organisation of Vine and Wine (OIV) [22]. All analyses were performed in triplicate (*n* = 3). Multiparameter measurements were carried out using a calibrated OenoFoss 2 analyzer (FOSS Analytical, Hillerød, Denmark) based on Fourier Transform Infrared (FTIR) spectroscopy [23,24]. Prior to analysis, samples were clarified by centrifugation or filtration to remove particulate matter (<25 µm). The following parameters were quantified: alcoholic strength (% *v*/*v*; OIV-MA-AS312-01), residual sugars (g L^−1^ glucose/fructose; OIV-MA-AS311-01), pH (OIV-MA-AS313-15), density (g cm^−3^; OIV-MA-AS2-01), total titratable acidity (g L^−1^ tartaric acid; OIV-MA-AS313-01), volatile acidity (g L^−1^ acetic acid; OIV-MA-AS313-02), L-malic acid (g L^−1^; OIV-MA-AS313-11), and L-lactic acid (g L^−1^; OIV-MA-AS313-12) [25]. All physicochemical analyses were completed within the same vintage year as the harvest to minimize compositional changes during storage.

### 2.4. Phenolic Compounds

The LC/MS analysis methodology was based on previously published analytical methods for phenolic compound analysis (ran on an Agilent 1100 VL Ion Trap mass spectrometer, Agilent Technologies, Santa Clara, CA, USA) [26,27,28,29,30,31]. Chromatographic separation was achieved on a Zorbax SB-C18 column (100 mm x 3.0 mm i.d., 3.5 µm particle size; Agilent Technologies, Santa Clara, CA, USA) maintained at 48 °C. The mobile phase consisted of 0.2% (*v*/*v*) acetic acid in water (Solvent A) and 0.2% (*v*/*v*) acetic acid in methanol (Solvent B). A gradient elution was applied as follows: 0–30 min, linear gradient from 3% to 70% B; followed by a re-equilibration step with 3% B for 4 min. The flow rate was set at 1 mL/min, and the injection volume was 1 µL. Mass spectrometry detection was performed in negative ionization mode using either MS (SIM mode) or MS/MS (MRM model) (Table S1, Supplemental Material). The ESI source parameters were set as follows: source voltage −2000 V; interface temperature 300 °C, nebulizing gas flow 3 L/min, heating gas flow 15 L/min (air); drying gas flow 15 L/min (nitrogen), desolvation line temperature 300 °C, heat block temperature 400 °C.

Quantitative analysis was performed by monitoring either the pseudo-molecular ion (in case of MS analysis) or specific fragmentation transitions in case of MS/MS analysis. The monitored mass ions and the retention time of the compounds are presented in Table S1, Supplemental material. Data acquisition and processing were carried out using LabSolutions 5.128 software (Shimadzu Corporation, Kyoto, Japan). The analytical method was validated regarding linearity, limit of detection (LOD), limit of quantification (LOQ), precision, and accuracy. Linearity was assessed in the 0.05–50 µg/mL range, LOD and LOQ were calculated based on a signal-to-noise ratio (S/N) of 5 and 10, respectively, and the calibration curve parameters are presented in Table S1, Supplementary Material.

### 2.5. Chromatic Parameters

Chromatic parameters were determined in the CIELab color space according to the methodology established by the International Commission on Illumination (CIE) [32]. The CIELab coordinates L* (lightness), a* (red–green axis), and b* (yellow–blue axis), together with chroma (C*ab), color intensity (I), and hue (N), were calculated from the visible absorption spectra of each sample. Spectra were recorded in the visible range (380–780 nm) with an Analytik Jena SPECORD^®^ 200 UV–VIS spectrophotometer (Analytik Jena GmbH & Co. KG, Jena, Germany) equipped with 1 cm optical path glass cuvettes and connected to an IBM-compatible PC. Color differences were evaluated using mathematical models implemented in the VINCOLOR program [33], which decompose any color difference into its constituent components of lightness, hue, and intensity according to the CIE color pattern.

### 2.6. Statistical Analysis

Experimental results are presented as the mean ± standard deviation (SD). Statistical analyses were conducted using XLSTAT 2019.2.2 (Addinsoft, Paris, France). Differences among samples were evaluated by one-way analysis of variance (ANOVA). The homogeneity of variances was assessed using Levene’s test. When significant differences were detected (*p* < 0.05), mean values were separated using Tukey’s Honestly Significant Difference (HSD) post hoc test at a significance level of 95%. For variables that did not satisfy the homogeneity-of-variance assumption, Welch’s ANOVA followed by Games–Howell pairwise comparisons was used. The one-way analyses were retained as secondary analyses to compare the individual experimental wines and generate the letter-based groupings reported in the tables; they were not used to estimate the independent effects of cultivar, harvest maturity, or maceration duration.

The effects of cultivar, harvest maturity, and maceration duration on the physicochemical and phenolic response variables were evaluated using a three-way fixed-effects ANOVA. The model included the main effects of cultivar, maturity, and maceration duration, all two-way interactions, and the cultivar × maturity × maceration duration interaction. The factorial analysis included the treatment combinations common to both cultivars and harvest maturity stages, corresponding to maceration durations of 0, 1, 3, and 6 months. Post-fermentation controls were excluded from this analysis because corresponding controls were unavailable at technological maturity, resulting in an incomplete factorial structure. Type III sums of squares were used to evaluate the model effects.

Model assumptions were evaluated using Levene’s test for homogeneity of variance and the Shapiro–Wilk test for residual normality. Residual sugar values were log10-transformed before factorial analysis to improve variance homogeneity. The transformation did not alter the presentation of the descriptive results, which are reported on the original scale. To reduce variance heterogeneity, TP, TPA, TFlav, and ellagic acid were log10-transformed, whereas total stilbenes and esculetin were transformed as log10(x + 0.001) because zero values were present. Following transformation, Levene’s test was nonsignificant for all phenolic response variables included in the factorial analysis. Variance heterogeneity persisted for some physicochemical responses, particularly volatile acidity and malic acid, and residual normality remained imperfect for several variables. Consequently, the corresponding factorial results were interpreted cautiously. Untransformed arithmetic means and standard deviations are presented in the tables to facilitate biological interpretation.

To investigate relationships among physicochemical parameters, phenolic compounds, phenolic indices, and color parameters, Pearson’s correlation coefficients (r) were calculated and visualized using a correlation heatmap. Correlations were considered statistically significant at *p* < 0.05. *p*-values were adjusted for multiple testing using the Benjamini–Hochberg false-discovery-rate procedure across all unique pairwise correlations. Associations were considered statistically supported at an adjusted *p*-value (q-value) of <0.05. Because this analysis was based on treatment-level observations and several variables were intrinsically or mathematically related, the correlations were considered exploratory and were not interpreted as evidence of independent causal relationships.

The multivariate structure of the dataset was explored using Principal Component Analysis (PCA) based on standardized variables. PCA was applied separately to grape skin phenolic fractions, grape seed phenolic fractions, and wine physicochemical and phenolic parameters to identify patterns of variation among samples and to determine the variables contributing most to sample differentiation. PCA was regarded as an exploratory descriptive analysis and not as a formal test of the effects of the experimental factors.

## 3. Results and Discussion

### 3.1. Effect of Extended Maceration on Wine General Physicochemical Parameters

The evolution of standard oenological parameters, organic acid profiles, and corresponding statistical indicators for all experimental wine variants are presented in Table 2. Significant differences were observed among the experimental wines for all evaluated parameters except density. These comparisons reflect the combined variation among cultivars, harvest maturity stages, and maceration treatments and do not, by themselves, isolate the independent effect of maceration duration.

The three-way factorial ANOVA showed significant main effects of cultivar and maceration duration on all evaluated physicochemical parameters except density (Table S3). The cultivar effect was significant for alcoholic strength, residual sugars, titratable acidity, volatile acidity, malic acid, and L-lactic acid (all *p* < 0.0001), as well as for pH (*p* = 0.008). Maceration duration significantly affected all these parameters (all *p* < 0.0001). Harvest maturity significantly affected alcoholic strength, residual sugars, titratable acidity, malic acid, L-lactic acid, and pH (all *p* < 0.0001), but not volatile acidity (*p* = 0.393). The cultivar × maturity interaction was nonsignificant for alcoholic strength (*p* = 0.164) and pH (*p* = 0.688), while the maturity × maceration interaction was nonsignificant only for pH (*p* = 0.106). The cultivar × maceration interaction was significant for all physicochemical responses other than density (all *p* ≤ 0.005), and the three-factor interaction was also significant for these responses (all *p* < 0.001). Density was not significantly affected by cultivar (*p* = 0.362), harvest maturity (*p* = 0.913), maceration duration (*p* = 0.991), or any interaction (all *p* ≥ 0.977). Because some model assumptions remained imperfect, these results were interpreted cautiously.

The alcoholic strength (AC) of the baseline control treatments representing immediate pressing without skin contact was 12.12% *v*/*v* for the SB variant (SBMDC) and 9.94% *v*/*v* for the FR variant (FRMDC). Prolonged maceration did not lead to a linear increase in ethanol concentration. Instead, a minor decrease in alcohol content occurred over the 6-month timeline for several treatments. For instance, SBMD6 decreased to 11.38% *v*/*v*. Because these vinifications were conducted in sealed stainless steel (inox) vessels, this cannot be attributed to evaporative headspace losses. Rather than chemical degradation, the primary hypothesis posits that ethanol depletion occurs via physical adsorption into the solid pomace matrix. Under this mechanism, the substantial volume of un-pressed grape pomace (3:2 *v*/*v* ratio) retained within the tanks is expected to act as an expansive biological filter [34]. Over extended contact times, the degrading cell wall structures of the grape skins and seed coats would progressively adsorb solvent components from the medium [35]. This process would effectively lock a fraction of the hydro-alcoholic phase within the solid pomace matrix, thereby lowering its measurable concentration in the free-run liquid phase [36].

**Table 2:** Evolution of general physicochemical parameters and organic acid profiles across experimental wine variants subjected to extended maceration.

**Samples**	**AS**	**RS**	**TA**	**VA**	**MA**	**LA**	**pH**	**D**
SBMDC	12.12 ± 0.14 ^h^	3.19 ± 0.03	6.16 ± 0.06 ^j^	0.17 ± 0.00	1.39 ± 0.01	0.34 ± 0.00 ^a^	2.98 ± 0.03 ^ab^	0.98964 ± 0.00757 ^a^
SBMD1	11.69 ± 0.10 ^fg^	0.72 ± 0.01	4.67 ± 0.05 ^cd^	0.39 ± 0.00	0.30 ± 0.00	1.08 ± 0.01 ^i^	3.28 ± 0.04 ^cde^	0.98987 ± 0.00968 ^a^
SBMD3	11.70 ± 0.10 ^g^	0.92 ± 0.01	4.86 ± 0.04 ^ef^	0.67 ± 0.01	0.32 ± 0.00	1.21 ± 0.01 ^lm^	3.28 ± 0.04 ^cde^	0.98925 ± 0.00736 ^a^
SBMD6	11.38 ± 0.13 ^ef^	0.53 ± 0.00	4.78 ± 0.05 ^cde^	0.65 ± 0.01	0.40 ± 0.00	1.23 ± 0.01 ^mn^	3.32 ± 0.04 ^def^	0.98951 ± 0.00989 ^a^
SBMFC	12.53 ± 0.13 ^i^	1.49 ± 0.01	6.46 ± 0.04 ^l^	0.21 ± 0.00	1.23 ± 0.01	0.49 ± 0.00 ^d^	3.01 ± 0.03 ^b^	0.98877 ± 0.00788 ^a^
SBMFCP	12.29 ± 0.07 ^hi^	1.18 ± 0.01	5.08 ± 0.06 ^gh^	0.26 ± 0.00	0.83 ± 0.01	0.92 ± 0.01 ^g^	3.29 ± 0.03 ^cde^	0.99008 ± 0.01050 ^a^
SBMF1	12.32 ± 0.11 ^hi^	1.18 ± 0.01	4.74 ± 0.05 ^cde^	0.36 ± 0.00	0.28 ± 0.00	1.03 ± 0.01 ^h^	3.32 ± 0.02 ^def^	0.98966 ± 0.01054 ^a^
SBMF3	12.16 ± 0.13 ^h^	1.01 ± 0.01	4.95 ± 0.04 ^fg^	0.65 ± 0.01	0.38 ± 0.00	1.24 ± 0.01 ^n^	3.39 ± 0.03 ^f^	0.98938 ± 0.00840 ^a^
SBMF6	12.24 ± 0.11 ^hi^	1.49 ± 0.01	5.13 ± 0.04 ^h^	0.68 ± 0.00	0.45 ± 0.01	1.20 ± 0.01 ^l^	3.36 ± 0.04 ^ef^	0.98904 ± 0.01137 ^a^
FRMDC	9.94 ± 0.09 ^c^	0.25 ± 0.00	6.32 ± 0.05 ^k^	0.10 ± 0.00	1.23 ± 0.01	0.42 ± 0.00 ^c^	2.90 ± 0.02 ^a^	0.99122 ± 0.01006 ^a^
FRMD1	9.36 ± 0.09 ^b^	0.18 ± 0.00	4.82 ± 0.04 ^ef^	0.20 ± 0.00	0.51 ± 0.00	1.17 ± 0.01 ^k^	3.20 ± 0.02 ^c^	0.99259 ± 0.00753 ^a^
FRMD3	9.25 ± 0.08 ^ab^	0.10 ± 0.00	4.32 ± 0.04 ^b^	0.42 ± 0.00	0.45 ± 0.01	1.21 ± 0.01 ^lm^	3.30 ± 0.03 ^def^	0.99218 ± 0.00673 ^a^
FRMD6	10.45 ± 0.06 ^d^	1.07 ± 0.01	4.79 ± 0.04 ^de^	0.67 ± 0.01	0.60 ± 0.01	1.14 ± 0.01 ^j^	3.37 ± 0.03 ^ef^	0.99146 ± 0.00961 ^a^
FRMFC	11.16 ± 0.11 ^e^	0.15 ± 0.00	5.88 ± 0.04 ^i^	0.18 ± 0.00	0.96 ± 0.01	0.39 ± 0.00 ^b^	3.02 ± 0.03 ^b^	0.99047 ± 0.01118 ^a^
FRMFCP	10.35 ± 0.06 ^d^	0.65 ± 0.00	4.65 ± 0.02 ^c^	0.14 ± 0.00	1.36 ± 0.01	0.60 ± 0.00 ^e^	3.29 ± 0.02 ^cde^	0.99255 ± 0.01039 ^a^
FRMF1	10.42 ± 0.09 ^d^	0.64 ± 0.01	4.37 ± 0.04 ^b^	0.27 ± 0.00	0.50 ± 0.00	0.83 ± 0.01 ^f^	3.31 ± 0.03 ^def^	0.99221 ± 0.00577 ^a^
FRMF3	9.02 ± 0.10 ^a^	0.10 ± 0.00	3.89 ± 0.04 ^a^	0.29 ± 0.00	0.40 ± 0.00	1.14 ± 0.01 ^j^	3.25 ± 0.03 ^cd^	0.99253 ± 0.01023 ^a^
FRMF6	10.41 ± 0.10 ^d^	1.09 ± 0.01	4.31 ± 0.03 ^b^	0.62 ± 0.00	0.42 ± 0.00	1.16 ± 0.01 ^jk^	3.38 ± 0.04 ^ef^	0.99141 ± 0.00515 ^a^
**Omnibus Test**	**One-Way ANOVA**	**Welch ANOVA**	**One-Way ANOVA**	**Welch ANOVA**	**Welch ANOVA**	**One-Way ANOVA**	**One-Way ANOVA**	**One-Way ANOVA**
F	375.929	3338.892	793.326	729.594	2521.217	4375.200	66.614	0.065
df	17, 36	17, 13.403	17, 36	17, 13.406	17, 13.409	17, 36	17, 36	17, 36
*p*-value	<0.0001	<0.0001	<0.0001	<0.0001	<0.0001	<0.0001	<0.0001	1.0000
Test for homoscedasticity (Levene)								
F	0.253	2.142	0.170	3.294	2.118	1.350	0.352	0.131
*p*-value	0.9981	0.027	0.9998	0.001	0.029	0.2206	0.9876	1.0000

AS—alcoholic strength by volume (% *v*/*v*); RS—residual sugar (g L^−1^); TA—total titratable acidity (g L^−1^, expressed as tartaric acid equivalents); VA—volatile acidity (g L^−1^, expressed as acetic acid equivalents); MA—malic acid (g L^−1^); LA—L-lactic acid (g L^−1^); D—density (g cm^−3^). Data are presented as means ± standard deviations. For variables satisfying the homogeneity-of-variance assumption, differences among individual wines were evaluated using one-way ANOVA followed by Tukey’s HSD test. For RS, VA, and MA, for which Levene’s test indicated unequal variances, Welch’s ANOVA followed by Games–Howell pairwise comparisons was used. Different lowercase superscript letters indicate significant Tukey pairwise differences at *p* < 0.05. Because exact compact-letter groupings could not be obtained for the Games–Howell comparisons, letters are not displayed for RS, VA, and MA. Residual sugars (RSs) were within the international threshold for dry wines (< 4 g L^−1^) in all experimental variants. The highest RS concentration was observed in the un-macerated control SBMDC (3.19 g L^−1^), whereas treatments subjected to extended maceration show lower residual sugar levels, reaching minimum values in FRMD3 and FRMF3. This greater sugar consumption suggests that prolonged contact with grape solids provided a continuous release of micronutrients and lipids from the grape tissues into the fermenting medium [37]. These components, particularly yeast survival factors such as unsaturated fatty acids and sterols, are essential for maintaining plasma membrane integrity and stress tolerance under adverse conditions [38]. Their availability likely helped maintain yeast viability throughout fermentation, enabling complete sugar consumption despite the low fermentation temperature (10–12 °C). Consistent with this high degree of dryness, wine density (D) remained statistically uniform among all variants, ranging from 0.98877 to 0.99259 g cm^−3^.

Baseline control variants exhibited high titratable acidity (6.16 g L^−1^ for SBMDC and 6.32 g L^−1^ for FRMDC) and low pH values (2.98 and 2.90, respectively). The introduction of skin and seed contact induced a systematic shift in the acid matrix, characterized by a steady decline in titratable acidity accompanied by a marked increase in pH. By month 6, for example, SBMD6 showed a reduced TA of 4.78 g L^−1^ and a concurrent pH increase to 3.32. This trend might result from the diffusion of mineral elements from grape skin cell walls [39]. Under this mechanism, prolonged pomace contact enables the progressive leaching of intracellular potassium ions (K^+^) into the hydroalcoholic phase. These cations then react with free tartaric acid to yield insoluble potassium bitartrate (KHC_4_ H_4_ O_6_) crystals, a precipitation reaction further favored by the low stabilization temperature (10–12 °C) [40]. Similar trends of pH elevation and tartrate precipitation have been reported in other studies on skin contact white wines, where extended pomace contact consistently reduces buffering capacity and organic acid content compared with immediate pressing [41,42].

Volatile acidity (VA) served as our primary indicator to monitor the microbial health of the wine throughout the extended skin contact phase. The control samples, which were pressed immediately without skin contact, showed minimal initial values of 0.17 g L^−1^ (SBMDC) and 0.10 g L^−1^ (FRMDC). As anticipated during prolonged exposure to grape pomace, volatile acidity increased, reaching a peak at 6 months of 0.65 g L^−1^ (SBMD6) and 0.62 g L^−1^ (FRMF6). This gradual rise in acetic acid is a common occurrence in long-term maceration. It can be attributed either to the slow leaching of bound volatile acids directly from the grape skins or to the limited metabolic activity of indigenous microflora within the pomace cap [43]. Importantly, all variants remained well below the maximum statutory threshold of 1.20 g L^−1^ volatile acidity for white wines, as specified in the updated Compendium of International Methods of Analysis of Wines and Musts (OIV-MA-AS313-02) [22]. These results shows that an extended maceration period of up to 6 months can be maintained without compromising the industrial hygiene or the overall chemical stability of the wine.

### 3.2. Profile and Evolution of Individual Phenolic Fractions Within the Wine Matrix

The continuous solid–liquid extraction during the 6-month maceration period fundamentally reorganized the individual phenolic families and altered the chemical composition of the wine matrix. The three-way ANOVA showed significant main effects of cultivar, harvest maturity, and maceration duration on TP, TPA, TFlav, total stilbenes, esculetin, and ellagic acid (all *p* < 0.0001). Significant two-way and three-way interactions were also observed for all these responses (Table S2). The only nonsignificant interaction was cultivar × maturity for TPA (*p* = 0.071). Therefore, the effects of harvest maturity and maceration duration were not uniform across cultivars and treatment combinations, and the individual wine means were interpreted in the context of these interactions.

#### 3.2.1. Evolution of Phenolic Fractions

Quantitative tracking of the primary architectural groups showed that flavonoids (TFlav) and phenolic acids (TPA) constituted the predominant fractions defining the overall extraction profile of the wine. In the control matrices, baseline values were extremely low, reflecting a typical immediate-press white vinification. For example, the direct-pressed FRMDC control yielded a minor 1.522 mg L^−1^ of TPA and a bare 0.098 mg L^−1^ of TFlav, while SBMDC showed 3.115 mg L^−1^ of TPA and 11.949 mg L^−1^ of TFlav (Table 3).

Maceration with the grape pomace initiated a rapid extraction phase within the first 30 days of solid–liquid contact. Within the technological maturity harvest series (MD), the SBMD1 variant reached 18.898 mg L^−1^ for total phenolic acids (TPA) and 222.574 mg L^−1^ for total flavonoids (TFlav) by month 1. This initial phase likely happens because grape cell walls quickly swell and break down upon contact with the grape juice [44,45]. As fermentation continues, the rising alcohol levels seem to drive the process further, with ethanol acting as a solvent that rapidly dissolves flavonoids and phenolic acids that would otherwise struggle to dissolve in plain water [12]. This accumulation continued steadily throughout the extended maceration phase. Peak concentrations were recorded at the 6-month threshold within the phenolic maturity harvest series (MF), specifically, SBMF6 achieved maximum values of 25.629 mg L^−1^ (TPA) and 312.804 mg L^−1^ (TFlav). This prolonged extraction driven by advanced fruit maturity ultimately yielded the highest overall total phenolic content (TP) observed in the study at 338.792 mg L^−1^.

Interestingly, wine harvested at technological maturity (MD) showed a noticeable decline in total flavonoids (TFlav) at month 6 (FRMD6, 201.75 mg L^−1^) compared to its month 3 peak (266.84 mg L^−1^). On the contrary, the phenolic maturity counterparts (MF) consistently maintained or increased their flavonoid yields over time, peaking heavily at month 6 (FRMF6, 260.938 mg L^−1^). This contrasting behavior suggests that grapes harvested at standard maturity (MD) may lack the advanced cell wall breakdown achieved through extended vine ripening. As a result, the extracted flavonoids are less stable in solution. Over six months of continuous skin maceration, these unstable compounds rapidly polymerized and precipitated as sediment [46]. Concurrently, the degrading pomace mass likely acted as a natural fining agent, re-adsorbing free flavonoids onto the settling solids [47].

These phase-partitioning profiles align cleanly with the extraction boundaries observed in dedicated white grape skin contact studies. As demonstrated by Darias-Martín et al. [14], non-flavonoid fractions achieve a rapid solid–liquid equilibrium, whereas flavonoids demand vastly extended contact periods to diffuse fully from the cellular pomace. However, extending this solid–liquid contact exposes the unshielded matrix to distinct late-stage risks. The eventual drop-off in total flavonoids captured here in the technologically mature variant (FRMD6) closely might mirror the degradation patterns reported by Olejar et al. [41] during long-term Sauvignon blanc macerations, where free, unprotected flavonoids dropped after peaking due to spontaneous protein cross-linking or localized chemical oxidation.

#### 3.2.2. Dynamics of Specific Phenolic Markers: Esculetin and Ellagic Acid

Trace phenolic markers, specifically the coumarin derivative esculetin and the polyphenol ellagic acid, displayed highly distinct extraction pathways during the trial. Both markers were virtually absent or detected at bare baseline values (0.000 to 0.064 mg L^−1^) in the direct-pressed controls (SBMDC and FRMDC). This can confirm that these two compounds serve as reliable indicators of extraction from solid grape pomace.

Individual phenolic markers showed distinct extraction patterns based on where they were located within the grape skin matrix. For instance, esculetin concentrations quickly peaked within the first 1 to 3 months across most variants, reaching values of 0.113 mg L^−1^ in SBMF1 and 0.177 mg L^−1^ in SBMFCP, before experiencing a notable decline toward month 6. This rapid early accumulation indicates that while esculetin is quickly solubilized from the easily accessible, outer cell layers of the grape skins, it remains chemically reactive once in solution. Over extended contact times, this free fraction likely degrades or binds into larger compounds, leading to the late-stage drop [14].

Ellagic acid accumulation followed a highly progressive and resilient trend across the experimental matrix. In contrast to pathways requiring months of skin contact, substantial concentrations were rapidly unlocked early in the timeline or immediately post-fermentation, peaking at 0.145 mg L^−1^ in the 1-month variant (SBMF1) and 0.149 mg L^−1^ in the control variant (FRMFCP), which was pressed directly upon the completion of alcoholic fermentation. Over the full six months, the steady presence of this phenolic acid confirms that the solid matrix undergoes a gradual, continuous breakdown. This slow release keeps concentrations stable, avoiding the rapid drops seen in more fragile, skin-bound compounds [48].

#### 3.2.3. Evolution of Major Phenolic Fractions Across Maceration Timelines

The evolution of total phenolics (TP), total flavonoids (TFlav), and total polyphenolic index (TPI) provides a global view of how skin contact and grape maturity react during extended maceration. Looking at the control groups (SBMDC, SBMFC, FRMDC, and FRMFC), it is clear that avoiding skin contact leaves white wines with very low phenolic baselines, typically yielding TP values under 26 mg L^−1^ and TPI values below 3.0. Once skin and pomace contact begins, these metrics surge dramatically. Over the six-month timeline, however, extraction does not follow a linear path; instead, the curves diverge based on maceration duration and grape maturity at harvest. For the technologically mature treatments (SBMD), extraction was aggressive and continuous. TP values rose steadily from 242.03 mg L^−1^ at one month (SBMD1) to a maximum of 304.62 mg L^−1^ at six months (SBMD6), an evolution closely matched by TFlav (222.57 mg L^−1^ rising to 279.68 mg L^−1^).

A different trend emerges in the FR series. In the technologically mature variant (FRMD), TP values actually hit a top and dropped significantly by the end of the timeline, falling from 286.25 mg L^−1^ at month 3 down to 221.20 mg L^−1^ at month 6. This late-stage loss is mirrored in its TFlav pool, which dropped from 266.84 mg L^−1^ to 201.75 mg L^−1^.

Interestingly, advanced phenolic maturity (MF) completely reconfigures extraction evolution, protecting the extracted compounds over time. The SBMF series achieved the highest overall values in the entire study, with TP peaking at 338.79 mg L^−1^ and TFlav reaching 312.80 mg L^−1^ at six months (SBMF6). More importantly, the fully ripe sample (FRMF6) avoided the late drop seen in the less-ripe wines, maintaining a stable total polyphenol level (282.48 mg L^−1^) at the six-month mark. This stability shows that ripening on the vine might alter grape cell walls, releasing protective soluble sugars (polysaccharides) alongside the flavonoids. These natural molecules form a shield around the flavonoids, preventing them from clumping together and settling out during long skin contact [49,50].

The Folin–Ciocalteu Index (FCI) demonstrated significant statistical variation across treatments (F = 716.29, *p* < 0.0001). Direct-pressed controls yielded the lowest values (1.734 for SBMDC and 2.122 for FRMDC), confirming that extended skin contact is necessary to elevate the matrix’s overall reducing capacity. In the technological maturity (MD) series, FCI values peaked at month 3 before experiencing a slight late-stage decline by month 6 (4.157 for SBMD6 and 5.675 for FRMD6). In contrast, the phenolic mature FRMF series maintained consistently higher values throughout, led by the pressed control FRMFCP (6.331) and FRMF1 (6.238), while FRMF6 preserved a strong index of 6.104 at the six-month mark. These findings might confirm that advanced harvest maturity helps maintain a stable phenolic profile during long-term maceration.

### 3.3. Flavonoid and Stilbene Extraction in Extended Skin Contact White Wines

The quantitative changes in flavan-3-ol monomers, dimers, and trimers across variants are presented in Table 4. In the control white wines (SBMDC and FRMDC), flavan-3-ol concentrations remained negligible, reflecting a standard white vinification profile. However, upon initiating the extended skin contact treatment characteristic of “orange” wines, the extraction evolution shifted significantly. In the SBMD series, monomeric subunits such as catechin and epicatechin rose sharply by month 1 (SBMD1), reaching 22.926 mg L^−1^ and 23.112 mg L^−1^, respectively. This accelerated initial extraction shows that low-molecular-weight flavonoids partition rapidly into the liquid phase during the early, highly fluid stages of prolonged maceration, driven by their high solubility in early hydro-alcoholic musts [14].

As maceration extended through six months, a distinct second extraction phase occurred, which was especially noticeable for complex oligomeric tannins. Procyanidin B2 and the trimeric procyanidin C1 exhibited continuous accumulation, maximizing in the six-month skin contact variant (SBMF6) at 91.709 mg L^−1^ and 10.205 mg L^−1^, respectively. This sustained release patterns the true nature of long-macerated white wines: while easily accessible skin monomers stabilize or slightly decline due to late-stage chemical rearrangement, complex oligomeric structures require multi-month skin and seed contact.

Compound extraction plateaued in the post-fermentation pressed control (SBMFCP) due to immediate separation from the pomace. Consequently, its catechin (23.026 mg L^−1^) and procyanidin B2 (47.682 mg L^−1^) concentrations remained below those of the one-month continuous maceration treatment SBMF1 (24.142 mg L^−1^ and 61.704 mg L^−1^).

When comparing the two grape cultivars under these extended skin contact conditions, Fetească regală displayed a distinct evolution fingerprint compared to Sauvignon blanc. While the ultimate accumulation of complex oligomers like procyanidin B2 was slightly more conservative in the FRMF “orange” wines (66.670 mg L^−1^ in FRMF6), its early-stage mass transfer of monomers was remarkably aggressive. For instance, FRMD1 achieved a catechin concentration of 28.168 mg L^−1^, outperforming the corresponding SB variant. These results suggest that FR grape skins may feature a weaker pectin matrix and greater cell wall porosity. Such a structural trait would naturally accelerate the early release of skin flavonoids into the fermenting juice, well before the slower, seed-derived tannins begin to dissolve [51].

The individual stilbene isomers followed a distinct, independent extraction pathway that highlights the skin-bound extraction limits of “orange” wines. Both trans-resveratrol and cis-resveratrol were entirely absent in the directly crushed white controls but emerged steadily during long-term maceration. Rather than showing a linear increase over the entire 6-month period, these phytoalexins frequently reached their maximum at intermediate stages, such as in SBMFCP where cis-resveratrol peaked at 0.113 mg L^−1^ and FRMD1 where trans-resveratrol reached 0.040 mg L^−1^, before hitting a plateau or dropping toward month 6. Because these individual stilbenes are localized almost exclusively in the superficial layers of the grape skin, they are completely liberated during the initial cell lysis of extended maceration. Over months of contact, these free monomers might undergo oxidative degradation or systematically adsorb onto settling yeast autolysates and insoluble pomace fragments, establishing a chemical equilibrium that defines the final polyphenolic signature of these extended skin contact white wines [52].

**Table 4:** Concentration of individual flavan-3-ols and stilbenes (mg L^−1^) across experimental wine variants subjected to extended maceration.

Samples	Gallocatechin	Catechin	Epicatechin	Procyanidin B3	Procyanidin B1	Procyanidin B4	Procyanidin B2	Procyanidin C1	Procyanidin A1	Procyanidin A2	Total Flavan-3-ols	trans-Resveratrol	cis-Resveratrol	Total Stilbenes
SBMDC	0.356 ^b^	3.057 ^b^	1.536 ^b^	0.879 ^ab^	3.661 ^b^	0.443 ^a^	1.997 ^ab^	0.020 ^a^	0.000 ^a^	0.000 ^a^	11.949 ^bc^	0.000 ^a^	0.000 ^a^	0.000 ^a^
SBMD1	1.090 ^def^	22.926 ^de^	23.112 ^h^	36.203 ^fg^	42.645 ^i^	32.281 ^b^	57.876 ^g^	6.380 ^h^	0.046 ^k^	0.015 ^c^	222.574 ^hi^	0.036 ^h^	0.052 ^f^	0.088 ^f^
SBMD3	1.103 ^ef^	22.805 ^d^	24.827 ^ij^	38.977 ^hi^	37.894 ^h^	37.582 ^d^	62.082 ^h^	6.699 ^i^	0.000 ^a^	0.015 ^c^	231.984 ^ij^	0.011 ^c^	0.073 ^h^	0.084 ^f^
SBMD6	1.267 ^fgh^	24.556 ^f^	24.893 ^ij^	46.733 ^lm^	49.277 ^k^	44.444 ^g^	79.967 ^k^	8.473 ^j^	0.042 ^j^	0.025 ^e^	279.677 ^l^	0.032 ^g^	0.064 ^g^	0.096 ^g^
SBMFC	0.472 ^bc^	4.440 ^c^	2.704 ^c^	2.233 ^b^	5.566 ^c^	1.492 ^a^	4.081 ^b^	0.228 ^a^	0.000 ^a^	0.000 ^a^	21.216 ^c^	0.006 ^b^	0.016 ^b^	0.022 ^b^
SBMFCP	1.266 ^fgh^	23.026 ^de^	21.453 ^d^	31.975 ^d^	42.870 ^i^	30.844 ^b^	47.682 ^e^	5.570 ^f^	0.054 ^l^	0.015 ^c^	204.755 ^fg^	0.064 ^j^	0.113 ^j^	0.177 ^j^
SBMF1	1.378 ^h^	24.142 ^ef^	23.334 ^h^	39.889 ^i^	48.757 ^k^	36.735 ^d^	61.704 ^h^	6.782 ^i^	0.037 ^i^	0.017 ^d^	242.775 ^j^	0.038 ^hi^	0.075 ^h^	0.113 ^i^
SBMF3	1.206 ^fgh^	24.842 ^fg^	27.284 ^hi^	43.036 ^j^	45.068 ^j^	41.524 ^e^	73.188 ^j^	6.753 ^i^	0.033 ^h^	0.000 ^a^	262.934 ^k^	0.013 ^c^	0.043 ^e^	0.056 ^d^
SBMF6	1.194 ^fg^	25.940 ^gh^	25.794 ^h^	52.598 ^n^	54.205 ^l^	51.056 ^h^	91.709 ^l^	10.205 ^k^	0.072 ^m^	0.031 ^f^	312.804 ^m^	0.022 ^e^	0.081 ^i^	0.103 ^h^
FRMDC	0.000 ^a^	0.086 ^a^	0.012 ^a^	0.000 ^a^	0.000 ^a^	0.000 ^a^	0.000 ^a^	0.000 ^a^	0.000 ^a^	0.000 ^a^	0.098 ^a^	0.000 ^a^	0.000 ^a^	0.000 ^a^
FRMD1	1.345 ^gh^	28.168 ^i^	21.343 ^fg^	37.236 ^gh^	29.617 ^g^	42.046 ^ef^	50.991 ^f^	4.642 ^e^	0.025 ^f^	0.000 ^a^	215.413 ^gh^	0.040 ^i^	0.042 ^e^	0.082 ^f^
FRMD3	0.962 ^cde^	31.261 ^k^	25.275 ^j^	48.205 ^m^	29.548 ^g^	59.087 ^i^	66.555 ^i^	5.902 ^g^	0.040 ^j^	0.000 ^a^	266.835 ^k^	0.025 ^f^	0.036 ^d^	0.061 ^d^
FRMD6	0.960 ^cde^	26.837 ^h^	20.352 ^f^	35.032 ^ef^	22.224 ^k^	44.475 ^g^	47.695 ^e^	4.157 ^d^	0.016 ^c^	0.000 ^a^	201.748 ^f^	0.012 ^c^	0.033 ^cd^	0.045 ^c^
FRMFC	0.016 ^a^	0.705 ^a^	0.450 ^a^	0.545 ^ab^	0.355 ^c^	0.676 ^a^	0.702 ^a^	0.072 ^a^	0.000 ^a^	0.000 ^a^	3.521 ^ab^	0.000 ^a^	0.000 ^a^	0.000 ^a^
FRMFCP	1.028 ^cde^	22.039 ^d^	15.965 ^d^	27.131 ^c^	17.270 ^d^	34.681 ^c^	30.913 ^c^	2.046 ^c^	0.019 ^d^	0.000 ^a^	151.092 ^d^	0.007 ^b^	0.036 ^d^	0.043 ^c^
FRMF1	1.251 ^fgh^	25.152 ^fg^	18.834 ^e^	33.311 ^de^	21.176 ^e^	43.692 ^fg^	40.616 ^c^	1.535 ^b^	0.013 ^b^	0.000 ^a^	185.580 ^e^	0.017 ^d^	0.032 ^c^	0.049 ^c^
FRMF3	1.123 ^ef^	29.685 ^jk^	23.697 ^h^	45.207 ^kl^	27.599 ^fg^	58.928 ^i^	62.925 ^hi^	5.429 ^f^	0.029 ^g^	0.000 ^a^	254.622 ^k^	0.030 ^g^	0.043 ^e^	0.073 ^e^
FRMF6	0.942 ^cd^	29.591 ^j^	23.153 ^h^	44.849 ^jk^	26.717 ^f^	63.422 ^j^	66.670 ^i^	5.559 ^f^	0.022 ^e^	0.013 ^b^	260.938 ^k^	0.007 ^b^	0.042 ^e^	0.049 ^c^

Values represent means (*n* = 3). Different lowercase letters within the same column indicate statistically significant differences according to Tukey’s HSD test (*p* < 0.05).

### 3.4. Interparametric Correlation Analysis of Acid Evolution, Phenolic Extraction, and CIELAB Color Evolution

**Figure 2:**
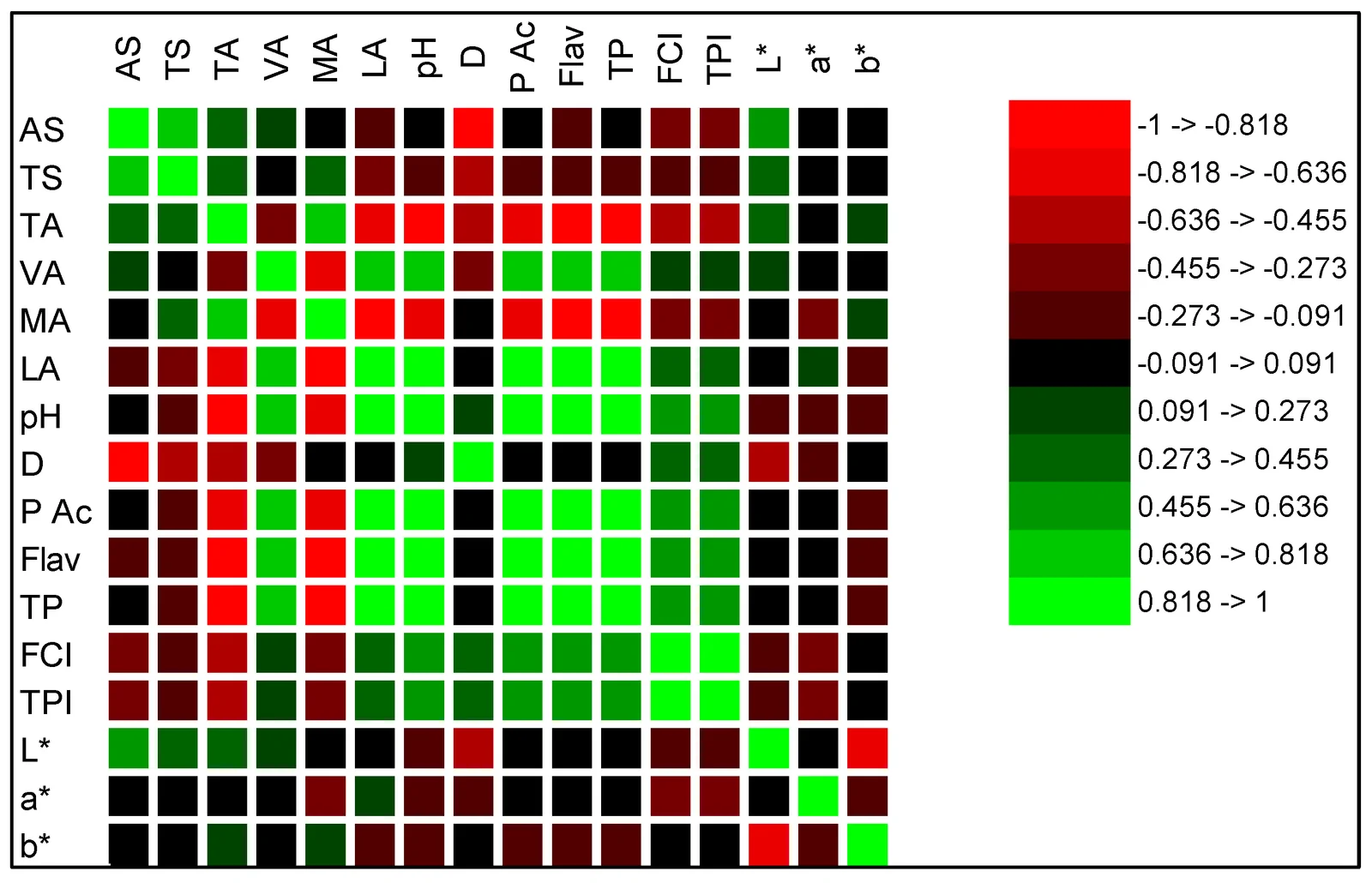
Pearson correlation heatmap showing exploratory associations among relationships between physicochemical parameters, phenolic compounds, and phenolic indices and CIELAB color parameters across the experimental wine treatments. Cells display Pearson correlation coefficients (r). Statistical support was evaluated after Benjamini–Hochberg correction across the 120 unique pairwise tests, using an adjusted *p*-value (q-value) threshold of <0.05. Correlations were not interpreted as evidence of causality.

To evaluate the relationships among basic enological parameters, HPLC-quantified phenolic classes, global spectrophotometric indices, and CIELAB color coordinates during extended skin contact, a comprehensive Pearson correlation analysis was performed (Figure 2). Because the analysis was based on a limited number of treatment groups and many variables differed systematically among treatments, the correlation coefficients were considered exploratory and were not interpreted as evidence of independent causal relationships.

After Benjamini–Hochberg correction, titratable acidity (TA) remained positively associated with malic acid (MA) (r = 0.725, q = 0.003). L-lactic acid (LA) was inversely associated with MA (r = −0.901, q < 0.001) and positively associated with pH (r = 0.847, q < 0.001) and volatile acidity (VA) (r = 0.774, q < 0.001). TA was inversely associated with both LA (r = −0.764, q = 0.001) and pH (r = −0.830, q < 0.001). These associations were consistent with coordinated variation in the organic-acid profile among the experimental treatments, although they did not independently demonstrate the underlying biochemical mechanisms. Alcoholic strength (AS) was inversely associated with density (D) (r = −0.919, q < 0.001).

Total phenolic acids (TPA), total flavan-3-ols (TFlav), and total phenols (TP) displayed strong positive intercorrelations (r = 0.975–1.000, all q < 0.001). These high coefficients were expected because TP was calculated from related phenolic components and should not be interpreted as independent biological associations. These HPLC-quantified phenolic parameters were positively associated with LA (r = 0.921–0.953, all q < 0.001) and pH (r = 0.919–0.942, all q < 0.001) and inversely associated with TA (r = −0.780 to −0.832, all q ≤ 0.001) and MA (r = −0.802 to −0.849, all q < 0.001). These relationships may partly reflect the common effects of cultivar, harvest maturity, and maceration duration on both phenolic and physicochemical variables and do not demonstrate that changes in organic acids directly caused phenolic extraction.

The Folin–Ciocalteu index (FCI) and total polyphenol index (TPI) were almost perfectly correlated (r = 1.000, q < 0.001). However, their correlations with the HPLC-derived phenolic variables were moderate (r = 0.469–0.480) and did not remain statistically supported after correction for multiple testing (q = 0.124–0.132). The CIELAB parameters showed limited associations with the measured phenolic fractions. The coefficients relating lightness (L*) to alcoholic strength (r = 0.491, q = 0.119) and density (r = −0.556, q = 0.055) did not remain statistically supported after Benjamini–Hochberg correction. In contrast, yellowness (b*) remained inversely associated with L* (r = −0.752, q = 0.002). These exploratory associations describe coordinated variation among the treatment groups but do not establish direct effects of phenolic variables on wine color.

**Figure 3:**
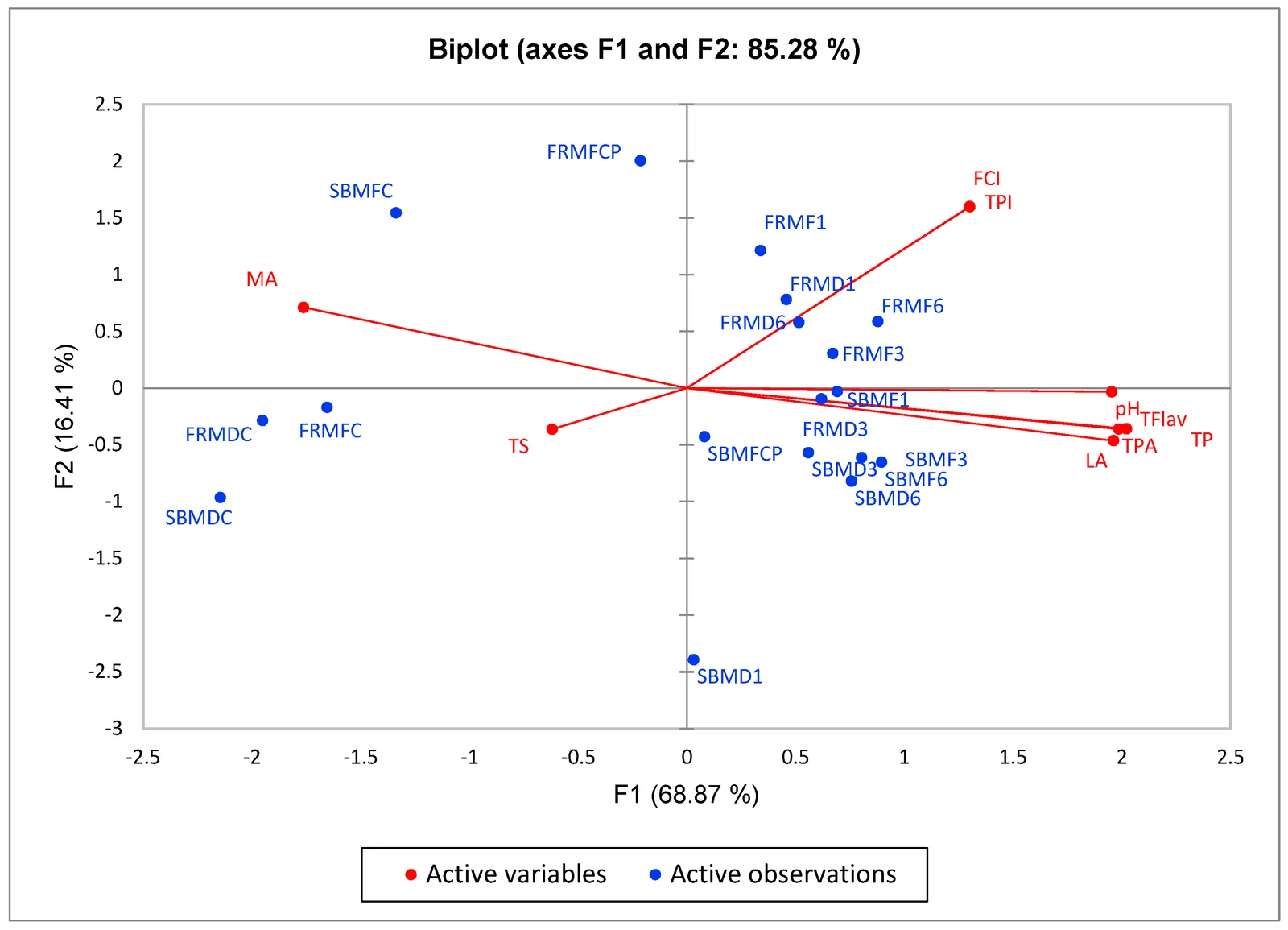
Principal Component Analysis (PCA) biplot (axes F1 and F2, explaining 85.28% of total variance) illustrating the relationship between active variables (red vectors) and wine samples (blue dots, active observations) across different cultivars (Sauvignon blanc and Fetească regală), harvest maturity stages, and maceration durations. MA = malic acid; LA = L-lactic acid; pH = matrix pH; TS = total sugars; FCI = Folin–Ciocalteu index; TPI = total polyphenol index; TPA = total phenolic acids; TFlav = total flavan-3-ols; TP = total phenols.

To further elucidate sample clustering and discrimination according to maceration duration, harvest maturity, and cultivar, PCA was performed on the active variables and observations (Figure 3). The first two principal components accounted for 85.28% of the total variance in the dataset, with F1 explaining 68.87% and F2 explaining 16.41%. The principal dimension F1 primarily represents the extent of skin contact and phenolic extraction; high positive loadings along F1 were driven by the HPLC-quantified phenolic classes (TP, TFlav, TPA), fermentation byproducts (LA), and matrix pH, which tightly vector together in the lower right quadrant. Conversely, MA and total sugars (TS) loaded strongly in the negative direction of F1, effectively separating the unmacerated control samples (SBMDC, FRMDC, SBMFC, FRMFC) situated on the left side of the biplot from their long-macerated counterparts. The global phenolic indices (FCI and TPI) loaded heavily on both positive F1 and positive F2, capturing overall polyphenol accumulation in the upper right quadrant. Along axis F2, sample distribution reflects cultivar-specific extraction traits and post-fermentative pressing treatments; for instance, early-pressed control samples (FRMFCP, SBMFC) and shorter macerations located higher along F2, whereas extended 3- to 6-month skin contact samples (SBMD3, SBMD6, SBMF3, SBMF6, FRMD3, FRMD6) clustered distinctly in the positive F1 region, underscoring the convergence toward a unified, highly phenolic hydroalcoholic matrix over multi-month pomace contact.

### 3.5. Solvent Partitioning and Acid–Base Reorganization

Rather than reflecting evaporation or microbial activity, the minor alcohol drop seen in sealed tanks during long maceration suggests that ethanol becomes trapped in the grape solids. Cell wall sugars likely bind water and ethanol at unequal rates, shifting skin porosity and locking alcohol inside the pomace [53,54], which would account for a lower final alcohol yield in extended skin contact wines [55].

The simultaneous drop in titratable acidity and increase in pH point toward a plausible solid–liquid extraction mechanism. Prolonged maceration likely accelerates the structural breakdown of grape skin cell walls, facilitating the passive diffusion of intracellular potassium (K^+^) ions into the liquid matrix [40]. In a hydroalcoholic solution, these cations would readily bind with free tartaric acid to form potassium bitartrate. Given the cold ambient conditions (10–12 °C), the solubility limit of these bitartrate salts was likely exceeded, promoting their physical crystallization and precipitation from the liquid phase [56,57].

The inverse relationship between malic acid depletion and phenolic accumulation points toward a shared extraction which might be driven by progressive cell wall degradation. As extended maceration breaks down the pectic and hemicellulosic framework of grape skins, it concurrently liberates bound polyphenols while accelerating the release and subsequent degradation of vacuolar organic acids. This structural nexus is strongly supported by significant three-way interactions across all primary acid parameters, including malic acid (F = 164.571, *p* < 0.0001), titratable acidity (F = 9.741, *p* < 0.001), and pH (F = 9.579, *p* < 0.001). These significant Cultivar × Maturity × Maceration (C × M × Mac) terms demonstrate that organic acid evolution does not proceed as a uniform background event. Instead, the rate of cell wall disassembly is dictated by cultivar-specific tissue architecture, harvest maturity, and contact duration. Ultimately, initial harvest maturity defines the structural susceptibility of the berry cell walls, whereas maceration time governs the degree of physical decay, jointly establishing the final equilibrium between phenolic richness, titratable acidity, and matrix stability [56,58].

### 3.6. Spatial Localization and Extraction Evolution of Flavan-3-Ols

The contrasting extraction patterns of flavan-3-ols reflect their distinct anatomical localization and molecular size within the berry. Skin-derived monomers are rapidly released during early maceration, whereas seed-derived oligomers require prolonged exposure to the evolving hydro-alcoholic solvent to diffuse from lignified seed coats [46,59]. This biphasic behavior is similar to the findings from other long-macerated white wine studies, which report an initial increase in catechin and epicatechin followed by a slower, continuous accumulation of procyanidin B2 and higher-order oligomers over several months of contact [15,41,60]. For example, in Malvazija istarska wines produced with prolonged maceration and barrel maturation, total phenols, color intensity, and antioxidant activity were markedly higher than in conventional white wines, with a clear enrichment in flavan-3-ols and flavonols. Similarly, in Minutolo and Verdeca, 6-month maceration strongly increased flavonoid levels and shifted the phenolic class balance toward flavonoids, in contrast to traditional white wines where hydroxycinnamic acids dominate. The eventual plateau or decline of free monomers during very extended contact has also been described in other “orange” wine trials and is commonly attributed to condensation and polymerization reactions that generate larger, less soluble pigmented tannins [4,15].

The multidimensional nature of these extraction trends may be further sustained by the complex interactions among cultivar, maturity, and maceration duration across major phenolic classes. Rather than operating in isolation, phenolic accumulation depends fundamentally on the close coupling of varietal background, harvest ripening stage, and contact duration. Specialized markers, such as ellagic acid and esculetin, exhibit similarly profound interactive effects, highlighting how distinct anatomical structures and developmental ripening stages might release rates and chemical transformations over time. This systemic interdependence confirms that the structural evolution of skin contact white wines is thoroughly multidimensional, driven by the combined influence of tissue architecture, maturity, and prolonged solvent exposure.

Cultivar differences further modulate this evolution. In our study, Sauvignon blanc exhibited a sustained release of dimeric and trimeric procyanidins over long maceration periods, in line with reports describing this variety as having more lignified, tannin-rich seed structures that support prolonged oligomer extraction. By contrast, Fetească regală showed a pronounced early release of skin-bound monomers, consistent with its more fragile skin pectin matrix and/or higher cell wall porosity. Similar cultivar-dependent patterns have been described in other skin contact white wines, where varieties with more porous or thinner skins display faster initial phenolic unloading, whereas those with denser seed tissues favor long-term oligomer accumulation [14,61].

### 3.7. Phenolic Stability and Wine Matrix Balance

The significant drop in individual flavonoids observed at the six-month mark in the technologically mature variants suggests that net extraction may reach a critical ceiling during extended macerations. A plausible explanation for this trend is that once fully released from the grape cells, these free monomeric and dimeric flavan-3-ols become increasingly vulnerable within the wine matrix [62]. Deprived of their natural cellular protection, these reactive molecules could undergo auto-oxidation, enzymatic browning, or re-adsorption over time. Furthermore, leftover solid grape fragments and spent yeast cell walls may act as a natural fining agent, potentially binding free flavonoids and pulling them out of solution through surface and chemical interactions [63,64], though direct tracking of degradation products would be required to verify this mechanism.

The fact that the phenolic maturity treatments resisted this late-stage drop points towards a possible protective macromolecular mechanism. Advanced ripening on the vine appears to reconfigure the grape skin cell walls, facilitating the natural release of soluble structural polysaccharides alongside the flavonoids. When extracted together, these native macromolecules may provide steric hindrance around the hydrophobic flavan-3-ols. They form a protective structural shield that limits the self-aggregation of grape tannins and prevents the individual flavonoids from binding together, oxidizing, or precipitating, keeping them stable in solution throughout the six-month maceration [65,66].

Data from the post-fermentation pressing controls offer further insight into these equilibrium boundaries. Separating the liquid must from the pomace bed immediately after fermentation stops mass transfer from the seeds, causing individual compound levels to plateau. However, the high color value recorded in the early-pressed Fetească regală control suggests that this prompt physical separation may successfully isolate highly colored monomeric pigments before they can be lost through late-stage precipitation or re-adsorption onto the lees [64]. Passing the three-month mark appears to alter the matrix dynamics entirely. The ongoing breakdown of deeper seed structures likely floods the wine with non-colored condensed tannins, shifting the orange wine toward a structurally dense system where color components may be stabilized through co-pigmentation and macromolecular binding rather than remaining as free monomers [62,67].

## 4. Conclusions

This study indicates that the physicochemical and phenolic characteristics of long-macerated white wines (“orange” wines) varied according to cultivar, harvest maturity, and maceration duration. The factorial analysis further indicated that the patterns associated with harvest maturity and maceration duration were not uniform across cultivars and treatment combinations. The observed extraction patterns were also consistent with differences in the molecular characteristics and localization of phenolic compounds within the grape berry. Skin-associated compounds, including flavan-3-ols, resveratrol, and esculetin, generally showed earlier changes, whereas procyanidins B2 and C1 and ellagic acid displayed a more gradual evolution during prolonged solid–liquid contact. Maceration for up to six months did not result in a uniform increase in all measured phenolic compounds. In some technologically mature treatments, the concentrations of individual monomeric phenolics decreased at the final sampling stage. These changes may be associated with oxidation, polymerization, precipitation, or adsorption onto lees and pomace fragments, although these mechanisms were not directly investigated in the present study. In contrast, some wines produced from grapes harvested at advanced phenolic maturity maintained higher concentrations of selected phenolic compounds during prolonged maceration. Differences between Fetească regală and Sauvignon blanc were also observed, but their magnitude and direction depended on harvest maturity and maceration duration. Finally, prolonged solid–liquid contact was associated with changes in the physicochemical composition of the wines, including lower titratable acidity and higher pH in several treatments. These observations are consistent with processes such as potassium extraction and potassium bitartrate precipitation during maceration; however, these mechanisms were not directly quantified in the present study. Intermediate maceration periods of 1–3 months were associated with favorable phenolic and color characteristics in several treatment combinations. However, longer maceration produced compound- and treatment-specific responses rather than a consistent improvement. Under the conditions evaluated in this study, maceration duration should therefore be selected according to cultivar, harvest maturity, and the desired wine composition. Further studies across additional vintages and experimental conditions, together with direct measurements of the proposed chemical mechanisms, are needed before a generally optimal maceration period can be established.

## Figures and Tables

**Table 1:** Comprehensive summary of sample codification and distinctive attributes.

No.	Sample Code	Grape Variety	Description
1	SBMDC	SB	Harvested at technological maturity, control
2	SBMD1	SB	Harvested at technological maturity, 1-month maceration
3	SBMD3	SB	Harvested at technological maturity, 3-month maceration
4	SBMD6	SB	Harvested at technological maturity, 6-month maceration
5	SBMFC	SB	Harvested at phenolic maturity, control
6	SBMFCP	SB	Harvested at phenolic maturity, post-pressing control
7	SBMF1	SB	Harvested at phenolic maturity, 1-month maceration
8	SBMF3	SB	Harvested at phenolic maturity, 3-month maceration
9	SBMF6	SB	Harvested at phenolic maturity, 6-month maceration
10	FRMDC	FR	Harvested at technological maturity, control
11	FRMD1	FR	Harvested at technological maturity, 1-month maceration
12	FRMD3	FR	Harvested at technological maturity, 3-month maceration
13	FRMD6	FR	Harvested at technological maturity, 6-month maceration
14	FRMFC	FR	Harvested at phenolic maturity, control
15	FRMFCP	FR	Harvested at phenolic maturity, post-pressing control
16	FRMF1	FR	Harvested at phenolic maturity, 1-month maceration
17	FRMF3	FR	Harvested at phenolic maturity, 3-month maceration
18	FRMF6	FR	Harvested at phenolic maturity, 6-month maceration

**Table 3:** Major phenolic fractions and global phenolic indices across experimental wine treatments.

Sample ID	TPA		TFlav		TS		Others				TP		FCI		TPI	
							Esculetin		Ellagic Acid							
SBMDC	3.115 ± 0.053 ^b^		11.949 ± 0.217 ^ab^		0.000 ± 0.000 ^a^		0.000 ± 0.000 ^a^		0.027 ± 0.001 ^a^		15.091 ± 0.271 ^ab^		1.734 ± 0.035 ^a^		1.73 ± 0.03 ^a^	
SBMD1	18.898 ± 0.318 ^f^		222.574 ± 4.454 ^gh^		0.088 ± 0.002 ^f^		0.103 ± 0.002 ^g^		0.363 ± 0.006 ^i^		242.026 ± 4.782 ^f^		1.626 ± 0.033 ^a^		1.63 ± 0.03 ^a^	
SBMD3	22.366 ± 0.433 ^i^		231.984 ± 4.479 ^hi^		0.084 ± 0.002 ^f^		0.069 ± 0.001 ^d^		0.381 ± 0.007 ^j^		254.884 ± 4.922 ^g^		4.456 ± 0.089 ^e^		4.46 ± 0.09 ^e^	
SBMD6	24.594 ± 0.427 ^j^		279.677 ± 5.042 ^l^		0.096 ± 0.002 ^g^		0.091 ± 0.002 ^f^		0.165 ± 0.003 ^g^		304.623 ± 5.476 ^j^		4.157 ± 0.083 ^d^		4.16 ± 0.08 ^d^	
SBMFC	4.629 ± 0.095 ^c^		21.216 ± 0.361 ^b^		0.022 ± 0.002 ^b^		0.017 ± 0.001 ^b^		0.049 ± 0.001 ^b^		25.933 ± 0.460 ^b^		5.219 ± 0.104 ^gh^		5.22 ± 0.10 ^gh^	
SBMFCP	22.234 ± 0.409 ^hi^		204.755 ± 3.367 ^ef^		0.177 ± 0.003 ^j^		0.169 ± 0.003 ^k^		0.362 ± 0.007 ^i^		227.697 ± 3.789 ^e^		3.990 ± 0.080 d		3.99 ± 0.08 ^d^	
SBMF1	21.577 ± 0.427 ^hi^		242.775 ± 4.558 ^i^		0.113 ± 0.003 ^i^		0.145 ± 0.003 ^ij^		0.457 ± 0.007 ^k^		265.067 ± 4.998 ^gh^		5.131 ± 0.103 ^gh^		5.13 ± 0.10 ^gh^	
SBMF3	24.307 ± 0.455 ^j^		262.934 ± 4.157 ^jk^		0.056 ± 0.002 ^d^		0.081 ± 0.001 ^e^		0.166 ± 0.003 ^g^		287.544 ± 4.618 ^i^		4.537 ± 0.091 ^e^		4.54 ± 0.09 ^e^	
SBMF6	25.629 ± 0.481 ^k^		312.804 ± 5.721 ^m^		0.103 ± 0.002 ^h^		0.101 ± 0.002 ^g^		0.155 ± 0.003 ^fg^		338.792 ± 6.209 ^k^		4.694 ± 0.094 ^ef^		4.69 ± 0.09 ^ef^	
FRMDC	1.522 ± 0.027 ^a^		0.098 ± 0.003 ^a^		0.000 ± 0.000 ^a^		0.047 ± 0.001 ^c^		0.064 ± 0.001 ^c^		1.731 ± 0.032 ^a^		2.122 ± 0.042 ^b^		2.12 ± 0.04 ^b^	
FRMD1	16.874 ± 0.265 ^e^		215.413 ± 3.805 ^fg^		0.082 ± 0.002 ^f^		0.136 ± 0.002 ^h^		0.147 ± 0.003 ^f^		232.652 ± 4.077 ^ef^		5.827 ± 0.117 ^jk^		5.83 ± 0.12 ^jk^	
FRMD3	19.133 ± 0.335 ^fg^		266.835 ± 4.675 ^k^		0.061 ± 0.002 ^d^		0.094 ± 0.002 ^f^		0.124 ± 0.002 ^e^		286.247 ± 5.016 ^i^		4.958 ± 0.099 ^fg^		4.96 ± 0.10 ^fg^	
FRMD6	19.247 ± 0.318 ^fg^		201.748 ± 3.488 ^e^		0.045 ± 0.002 ^c^		0.084 ± 0.002 ^e^		0.074 ± 0.001 ^c^		221.198 ± 3.811 ^e^		5.675 ± 0.114 ^ij^		5.68 ± 0.11 ^ij^	
FRMFC	2.390 ± 0.044 ^a^		3.521 ± 0.060 ^a^		0.000 ± 0.000 ^a^		0.050 ± 0.001 ^c^		0.051 ± 0.001 ^b^		6.012 ± 0.106 ^a^		2.522 ± 0.050 ^c^		2.52 ± 0.05 ^c^	
FRMFCP	15.514 ± 0.297 ^d^		151.092 ± 2.992 ^c^		0.043 ± 0.002 ^c^		0.149 ± 0.003 ^j^		0.124 ± 0.002 ^e^		166.922 ± 3.296 ^c^		6.331 ± 0.127 ^l^		6.33 ± 0.13 ^l^	
FRMF1	16.415 ± 0.297 ^de^		185.580 ± 3.309 ^d^		0.049 ± 0.002 ^c^		0.142 ± 0.003 ^hi^		0.209 ± 0.005 ^h^		202.395 ± 3.616 ^d^		6.238 ± 0.125 ^l^		6.24 ± 0.12 ^l^	
FRMF3	20.125 ± 0.337 ^g^		254.622 ± 4.863 ^j^		0.073 ± 0.002 ^e^		0.101 ± 0.002 ^g^		0.074 ± 0.002 ^c^		274.995 ± 5.206 ^hi^		5.414 ± 0.108 ^hi^		5.41 ± 0.11 ^hi^	
FRMF6	21.300 ± 0.377 ^h^		260.938 ± 4.268 ^jk^		0.049 ± 0.002 ^c^		0.107 ± 0.002 ^g^		0.086 ± 0.002 ^d^		282.480 ± 4.651 ^i^		6.104 ± 0.122 ^kl^		6.10 ± 0.12 ^kl^	
ANOVA ONE-WAY																
	TPA		TFlav		TS		E		Eac		TP		FCI		TPI	
	F	Pr > F	F	Pr > F	F	Pr > F	F	Pr > F	F	Pr > F	F	Pr > F	F	Pr > F	F	Pr > F
Analysis of variance	1616.438	<0.0001	2060.969	<0.0001	1460.366	<0.0001	1374.829	<0.0001	3338.389	<0.0001	2017.512	<0.0001	716.288	<0.0001	733.870	<0.0001
Test for homoscedasticity of the residuals	0.711	0.771	0.884	0.595	0.708	0.774	0.641	0.835	1.273	0.265	0.862	0.618	0.358	0.986	0.386	0.980

TPA—Total phenolic acids (mg L^−1^); TFlav—Total flavonoids (mg L^−1^); TS—Total stilbenes (mg L^−1^); TP—Total phenolics (mg L^−1^); FCI—Folin–Ciocalteu Index; TPI—Total Polyphenol Index. Different lowercase letters indicate significant differences among the named wines according to the secondary one-way ANOVA followed by Tukey’s HSD test (*p* < 0.05). These letter-based comparisons should not be interpreted as estimates of the independent effects of cultivar, maturity, or maceration duration. The corresponding factorial effects and interactions are presented in Table S2.

## Data Availability

The original contributions presented in this study are included in the article and the Supplementary Materials. The raw data supporting the conclusions of this study will be made available by the corresponding authors upon reasonable request.

## Supplementary Materials

The following supporting information can be downloaded at https://www.mdpi.com/article/10.3390/foods15183334/s1, Table S1: Individual phenolic compounds identified and quantified in Fetească regală and Sauvignon blanc white wines subjected to extended maceration across distinct harvest maturities; Table S2: Three-way factorial ANOVA for the effects of cultivar, harvest maturity, and maceration duration on phenolic variables; Table S3: Type III fixed-effects three-way ANOVA for the effects of cultivar, harvest maturity, maceration duration, and their interactions on wine physicochemical parameters.
