# Supplementary material for: Phenolic Evolution During Extended Skin Contact: Effects of Harvest Maturity on “Orange” Wine Composition and Color
Source: Foods. 2026 Sep 20;15(18):3334. doi: 10.3390/foods15183334 (PMC13606652; doi:10.3390/foods15183334)
Supplement: Supplementary file 1 [file foods-15-03334-s001.zip › foods-4519312-supplementary.pdf]

Table S1. Individual phenolic compounds identified and quantified in Fetească regală and Sauvignon blanc white wines subjected to extended maceration across distinct harvest maturities.

No.	Compound name	Retention time (min)	MS analysis type	Precursor ion, *m/z*	Calibration curve		
					Slope	Intercept	Correlation coefficient
1	Gallocatechin	2.84	MRM	305.066	167646	77874	0.9964
2	Procyanidin B3	4.75	MRM	577.134	119245	21579	0.9981
3	Procyanidin B1	5.20	MRM	577.134	103592	-13135	0.9978
4	Procyanidin B4	6.15	MRM	577.134	127379	-28968	0.9928
5	Procyanidin B2	7.40	MRM	577.134	103152	2282	0.9978
6	Catechin	5.81	SIM	289.071	266920	69348	0.9988
7	Esculetin	6.68	SIM	177.018	1435952	-253651	0.9990
8	Epicatechin	8.89	SIM	289.071	334620	152494	0.9995
9	Procyanidin C1	9.35	SIM	865.198	92509	33249	0.9868
10	Procyanidin A1	9.77	SIM	575.119	176974	46853	0.9958
11	Procyanidin A2	12.35	SIM	575.119	140502	-6686	0.9984
12	trans-Resveratrol	15.47	MRM	227.070	115287	94503	0.9844
13	cis-Resveratrol	18.16	MRM	227.070	119728	-1070	0.9992

Table S2. Three-way factorial ANOVA for the effects of cultivar, harvest maturity, and maceration duration on phenolic variables.

**Dependent variable**	**Effect**	**F**	**p-value**
log10(TP)	Cultivar	11029.831	<0.0001
	Maturity	2722.356	<0.0001
	Maceration	113390.121	<0.0001
	Cultivar × Maturity	117.612	<0.0001
	Cultivar × Maceration	6742.021	<0.0001
	Maturity × Maceration	1655.669	<0.0001
	Cultivar × Maturity × Maceration	418.237	<0.0001
log10(TPA)	Cultivar	3743.411	<0.0001
	Maturity	876.297	<0.0001
	Maceration	38914.417	<0.0001
	Cultivar × Maturity	3.476	0.071
	Cultivar × Maceration	572.054	<0.0001
	Maturity × Maceration	301.716	<0.0001
	Cultivar × Maturity × Maceration	24.691	<0.0001
log10(TFlav)	Cultivar	27801.874	<0.0001
	Maturity	10756.429	<0.0001
	Maceration	159812.960	<0.0001
	Cultivar × Maturity	3929.582	<0.0001
	Cultivar × Maceration	21272.517	<0.0001
	Maturity × Maceration	8527.006	<0.0001
	Cultivar × Maturity × Maceration	5054.796	<0.0001
log10(Total stilbenes + 0.001)	Cultivar	4926.062	<0.0001
	Maturity	1252.100	<0.0001
	Maceration	31318.535	<0.0001

	Cultivar × Maturity	1731.456	<0.0001
	Cultivar × Maceration	1077.878	<0.0001
	Maturity × Maceration	1697.104	<0.0001
	Cultivar × Maturity × Maceration	1699.141	<0.0001
log10(Esculetin + 0.001)	Cultivar	11628.907	<0.0001
	Maturity	5513.756	<0.0001
	Maceration	23000.177	<0.0001
	Cultivar × Maturity	3422.571	<0.0001
	Cultivar × Maceration	7931.725	<0.0001
	Maturity × Maceration	2511.950	<0.0001
	Cultivar × Maturity × Maceration	2773.552	<0.0001
log10(Ellagic acid)	Cultivar	7199.193	<0.0001
	Maturity	40.037	<0.0001
	Maceration	15044.661	<0.0001
	Cultivar × Maturity	13.403	0.000898
	Cultivar × Maceration	2919.968	<0.0001
	Maturity × Maceration	1298.258	<0.0001
	Cultivar × Maturity × Maceration	475.039	<0.0001

Three-way fixed-effects ANOVA evaluating the effects of cultivar, harvest maturity, maceration duration, and their interactions on phenolic response variables. Post-fermentation controls were excluded from the factorial analysis because corresponding controls were not available across the complete experimental design. Type III sums of squares were used. Given the experimental structure and the results of the assumption tests, the factorial effects were interpreted cautiously.

Table S3. Type III fixed-effects three-way ANOVA for the effects of cultivar, harvest maturity, maceration duration, and their interactions on wine physicochemical parameters.

**Parameter**	**Scale**	**Cultivar F (p-value)**	**Maturity F (p-value)**	**Maceration F (p-value)**	**C×M F (p-value)**	**C×Mac F (p-value)**	**M×Mac F (p-value)**	**C×M×Mac F (p-value)**
AS	Raw	4314.708 (<0.0001)	316.698 (<0.0001)	150.985 (<0.0001)	2.032 (0.164)	95.453 (<0.0001)	32.373 (<0.0001)	46.357 (<0.0001)
RS	log10	336511.754 (<0.0001)	7467.328 (<0.0001)	40438.078 (<0.0001)	19.539 (<0.001)	66874.526 (<0.0001)	19238.156 (<0.0001)	6323.640 (<0.0001)
TA	Raw	915.000 (<0.0001)	96.403 (<0.0001)	3939.970 (<0.0001)	670.043 (<0.0001)	146.266 (<0.0001)	6.751 (0.001)	9.741 (<0.001)
VA	Raw	7956.750 (<0.0001)	0.750 (0.393)	22490.750 (<0.0001)	18.750 (<0.001)	1968.750 (<0.0001)	388.750 (<0.0001)	294.750 (<0.0001)
MA	Raw	438.857 (<0.0001)	1441.714 (<0.0001)	42602.857 (<0.0001)	829.714 (<0.0001)	2260.571 (<0.0001)	678.286 (<0.0001)	164.571 (<0.0001)
LA	Raw	324.000 (<0.0001)	256.000 (<0.0001)	22083.333 (<0.0001)	676.000 (<0.0001)	23.333 (<0.0001)	475.333 (<0.0001)	204.667 (<0.0001)
pH	Raw	8.067 (0.008)	30.750 (<0.0001)	342.994 (<0.0001)	0.165 (0.688)	5.189 (0.005)	2.213 (0.106)	9.579 (<0.001)
D	Raw	0.854 (0.362)	0.012 (0.913)	0.034 (0.991)	0.001 (0.977)	0.014 (0.998)	0.007 (0.999)	0.001 (1.000)

Three-way fixed-effects ANOVA evaluating the effects of cultivar, harvest maturity, maceration duration, and their interactions on physicochemical response variables. The analysis was restricted to

the complete factorial subset of the experimental design. Post-fermentation controls were excluded because corresponding controls were not available across all combinations of cultivar and harvest maturity. Type III sums of squares were used. Because model assumptions remained imperfect for some physicochemical responses, the corresponding factorial effects were interpreted cautiously.
